# Antiadhesive Properties of Arabinogalactan Protein from *Ribes nigrum* Seeds against Bacterial Adhesion of *Helicobacter pylori*

**DOI:** 10.3390/molecules19033696

**Published:** 2014-03-24

**Authors:** Jutta Messing, Michael Niehues, Anna Shevtsova, Thomas Borén, Andreas Hensel

**Affiliations:** 1 Institute of Pharmaceutical Biology and Phytochemistry, University of Münster, D-48149 Münster, Germany; E-Mails: jutta.messing@uni-muenster.de (J.M.); m_niehues@hotmail.com (M.N.); 2 Department of Medical Biochemistry and Biophysics, Umeå University, Umeå, SE-901 87, Sweden; E-Mails: anna.shevtsova@medchem.umu.se (A.S.); thomas.boren@medchem.umu.se (T.B.)

**Keywords:** *Ribes nigrum* L., adhesion, antiadhesion, arabinogalactan protein, BabA, fibronectin, *Helicobacter pylori*, virulence factors

## Abstract

Fruit extracts from black currants (*Ribes nigrum* L.) are traditionally used for treatment of gastritis based on seed polysaccharides that inhibit the adhesion of *Helicobacter pylori* to stomach cells. For detailed investigations an arabinogalactan protein (F2) was isolated from seeds and characterized concerning molecular weight, carbohydrate, amino acid composition, linkage, configuration and reaction with β-glucosyl Yariv. Functional testing of F2 was performed by semiquantitative *in situ* adhesion assay on sections of human gastric mucosa and by quantitative *in vitro* adhesion assay with FITC-labled *H. pylori* strain J99 and human stomach AGS cells. Bacterial adhesins affected were identified by overlay assay with immobilized ligands. ^125^I-radiolabeled F2 served for binding studies to *H. pylori* and interaction experiments with BabA and SabA. F2 had no cytotoxic effects against *H. pylori* and AGS cells; but inhibited bacterial binding to human gastric cells. F2 inhibited the binding of BabA and fibronectin-binding adhesin to its specific ligands. Radiolabeled F2 bound non-specifically to different strains of *H. pylori*; and to BabA deficient mutant. F2 did not lead to subsequent feedback regulation or increased expression of adhesins or virulence factors. From these data the non-specific interactions between F2 and the *H. pylori* lead to moderate antiadhesive effects.

## 1. Introduction

Black currant (*Ribes nigrum* L. Grossulariaceae), also known as Cassis, is a deciduous shrub, reaching 1 to 2 m height, native to Northern and Central Europe as well as to Asia and widely cultivated in North America. The berry fruits, containing unsaturated fatty acids, anthocyanidins, flavonols, pectins, fruit acids, invert sugar and polysaccharides, are widely used in food technology, but also in traditional medicine for stomach pain, wound healing and inflammation in the oral cavity [1]. *R. nigrum* seeds occur in great quantities as by-products from juice and jam production and contain about 27% to 33% of fatty oil [2,3]. During the last years the seeds of black currants have been attracting attention for use in cosmetics and dermatology for skin regeneration and neurodermitis [4]. Beside the unsaturated fatty oil, seed polysaccharides also seem to influence skin cell physiology [4]. Recently the polysaccharide fraction has been described to reveal antiadhesive activity against *H. pylori* [5]. This finding rationalizes the traditional medical use of black currant fruit extracts as a foodstuff to treat gastric irritation. 

Preparations with antiadhesive properties against pathogens may be interesting tools for future medical developments, as they interact with the surface proteins of pathogens that have not yet been pinpointed as molecular targets [6]. Antiadhesive preparations cannot cure an acute infection, but might be used after eradication therapy to inhibit recurrence by preventing recolonization of the human stomach. Historically, the identification of antiadhesive compounds against *H. pylori* has been based on the initial finding of antiadhesive properties of 3'-sialyllactose [7], but unfortunately, this compound failed to prevent bacterial colonizaton of human stomach in a preliminary clinical study [8], likely owing to degradation of the compound under physiological conditions in the stomach. The search for additional antiadhesive compounds has yielded peptides [9], polyphenols [10,11], *N*-phenylpropenoyl-L-amino acid amides [12], and polysaccharides [5,13,14,15] that interact with bacterial outer membrane proteins (OMPs). The clinical and economic development of such antiadhesives is still underrepresented, in many cases because it is economically difficult to obtain sufficient amounts of these natural products at reasonable prices. Therefore, there is still a long way to go towards the development of registered antiadhesive drug products [6].

Recognition of and adhesion to epithelial cells by *H. pylori*, as well as the persistence of *H. pylori* in the stomach, is mainly mediated by several OMPs from the *Helicobacter* outer membrane protein (*hop*) group. Intensively studied members of the *hop* group include the blood group antigen binding adhesin (BabA, also known as HopS) and the sialic acid binding adhesin (SabA, also known as HopP). BabA interacts with fucosylated oligosaccharide structures present in H-1 and Lewis^b^ (Le^b^) blood group antigens [16,17]. BabA is also centrally involved in *H. pylori* binding to MUC5AC and MUC5B, even in non-secreting individuals that either lack an α-(1,2)-fucosyltransferase (and are therefore not able to express Le^b^ in high amounts) or lack Le^b^, and thereby acts as an important factor for initial colonization [18].

Furthermore, antigens such as sialyl-Lewis^a^ and sialyl-Lewis^x^, which are predominantly expressed in inflamed gastric tissue, interact with and bind to SabA [19]. Such fucosylated and sialylated antigens favor the colonization of *H. pylori* to the gastric mucosa, and might even promote the chronicity of infection once gastritis is established [19]. SabA is also the hemagglutinin responsible for sialic acid-dependent hemagglutination [20].

The adherence-associated lipoproteins (AlpA and AlpB, also known as HopB and HopC), outer inflammatory protein (OipA, also known as HopH), and HopZ are also associated with bacterial adhesion [21,22,23,24]. However, corresponding receptors for AlpA/B, OipA, and HopZ have not yet been identified. Another bacterial adhesin known as HpaA, a subunit of N-acetylneuraminyllactose-binding fibrillar hemagglutinin, can be blocked by the glycoprotein fetuin and 3'-sialyllactose. Exogenous 3'-sialyllactose can even reverse hemagglutination, and can detach adherent *H. pylori* from gastric cells [21]. In addition, interactions between *H. pylori* and extracellular matrix proteins such as laminin, fibronectin, and type IV collagen have been described to function as receptors in the gastric region [25,26,27]. With regard to these highly complex interactions and the importance of *H. pylori* adherence for the development of its pathogenicity, a precise comprehension of an inhibition mechanism for new antiadhesive compounds is essential. The life-long eradication of *H. pylori* by antibiotics is becoming increasingly problematic because of increasing antibiotic resistanceand the fact that the development of a prophylaxis by vaccination is challenging and still in progress [28]. Antiadhesive compounds could provide a preventive, cytoprotective strategy to control *H. pylori* colonization, especially to prevent its recurrence after antibiotic eradication therapy.

Several virulence factors have been elucidated to mediate pathogenicity and disease outcome of *H. pylori* infection. Aside from the flagella (which are important for mobility) and urease (which is necessary for acid tolerance), adhesins also play an important role: in addition to maintaining *H. pylori* colonization and persistence, adhesins also direct progression of the infection and incidence of the disease. Other virulence factors include the cag pathogenicity island (cagPAI) and vacuolating cytotoxin (VacA). Despite all knowledge about *H. pylori* attachment to the gastric epithelium, the associations and interactions between individual virulence factors and adhesion remain controversial. Therefore, we studied the hypothetical interaction of *R. nigrum* seed polysaccharides as an antiadhesive agent and its influence on the mRNA expression of several OMPs, as well as virulence factor encoding genes *ureA*, *ureI*, *fucT*, *cagA*, *cagα*, *cagL*, and *vacA*.

## 2. Results and Discussion

### 2.1. Isolation and Characterization of Arabinogalactan Protein F2 from R. nigrum Seeds

**Figure 1:**
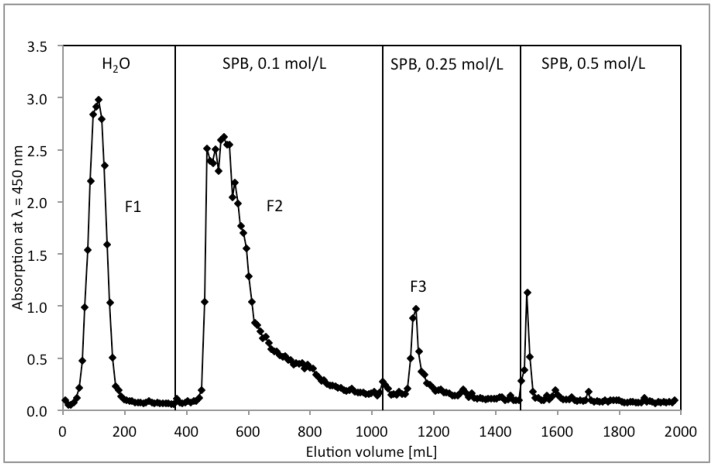
Chromatographic separation of raw polysaccharides (RPS) from *Ribes niger* seeds by anion exchange on DEAE-Sephacel^®^ by step gradient of water, 0.1, 0.25 and 0.5 mol/L SPB. Determination of the carbohydrate content of the eluent was performed by resorcinol sulfuric acid test (λ = 450 nm).

From the defatted seeds of *R. nigrum* raw polysaccharides RPS (yield 1.1%) were obtained by aqueous extraction. RPS was furtheron fractionated by ion exchange chromatography (Figure 1). Neutral polysaccharides were eluted with water and acidic polymers by increasing ion strength. The main fraction F2, eluting at 0.1 mol/L sodium phosphate buffer, was isolated in a yield of 0.3% related to the starting material. 

**Figure 2:**
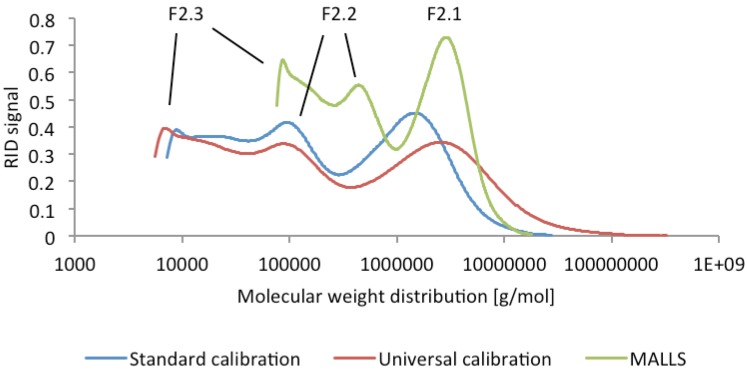
Molecular weight distribution of F2 determined by HP-SEC, according to standard calibration, universal calibration and multi angle light scattering (MALLS), indicating the presence of three subfractions F2.1, F2.2 and F2.3.

F2 reacted strongly positive with β-D-glucosyl Yariv reagent [29] and was assessed to be an arabinogalactan protein. By gel permeation chromatography F2 was shown to consist of a set of three subfractions F2.1 to F2.3. On GPC with Superose™6 stationary phase under low pressure conditions molecular weights of ~1,000, 107 and 50 kDa were determined. HP-SEC also indicated a similar peak pattern (Figure 2). 

Depending on the calibration method different MWs were calculated: For the standard calibration with pullulans molecular weights of 2,000, 120 and 21 kDa were determined, the universal calibration by viscosimetry gave 1,400, 100 and 9 kDa and the application of the data of multi angle light scatter measurement for the calculation by Zimm equation resulted in a MW of 1,000, 100 and 16 kDa, depending on the refractive index increment dn/dc. The MW dispersity Đ_M_ [30] was calculated by standard calibration measurement with 2.1, 1.3, and 1.3 for the three subfractions, by viscosimetry to be 7.4, 1.4, 1.2 and by light scattering a Đ_M_ to be 1.2, 1.1 and 1.0. 

SDS-PAGE of F2, followed by staining with Yariv reagent revealed two smearing high molecular bands, which were correlated to the subfractions F2.1 and F2.3, while in the range between 10 to 60 kDa no staining was visible. F2 contained 23% ± 5% uronic acids [30] and 1.03% ± 0.1% of polypeptides according determination by Bradford assay. By using HPAEC-PAD quantification of amino acid after hydrolysis against external standard calibration as determined by 3 independent quantifications 2.1% of polypeptides were determined. The sugar composition and the respective linkage parameters of F2 polysaccharides are summarized in Table 1, indicating the main structural features of a type II arabinogalactan with a (1,3)(1,3,6)-galactose backbone, bearing single branching units of 1-arabinose or short (1,6)-galactose side chains with 1,5-linked arabinose residues attached. 

**Table 1:** Carbohydrate and protein composition (mol%) of F2 from *R. nigrum*: monosaccharide composition as determined by HPAEC-PAD after TFA hydrolysis, results of linkage analysis after methylation of carboxyl-reduced material and GC-MS identification; quantitative amino acid distribution in Y2 as determined by HPAEC-PAD (mol %). tr.: traces, n.d. = not detected.

	Amount [%]	Carbohydrate	HPAEC- PAD	Linkage type	Amount GC-MS
			(mol %)		(mol %) *
Neutral carbohydrates	76	d-Gal	28.3	1-*p*	0.3
				1,3-*p*	13.5
				1,4-*p*	0.1
				1,6-*p*	0.3
				1,3,6-*p*	14.1
		l-Ara	24.5	1-*f*	4.2
				1,2-*f*	0.3
				1,5-*f*	10.9
				1,2,5-*f*	8.6
				1,3,5-*f*	0.5
		d-Xyl	15.7	1-*p*	9.2
				1,2-*p*	6.5
		d-Man	3.5	1,2-*p*	0.1
				1,4-*p*	1.0
				1,4,6-*p*	2.4
		l-Rha	2.4	1-*p*	tr.
				1,2,4-*p*	2.4
		d-Glc	1.0	1-*p*	0.4
				1,4-*p*	0.6
		l-Fuc	0.6	1-*p*	0.6
Uronic acids	23 ****	d-GalA	16.0	1,4-*p*	16.0
		d-GlcA	7.0	1-*p*	1.4
				1,4-*p*	5.6
Protein	1.0 ** 2.1***	**Amino acid**		**[mol %]**	
		Histidine		12.1	
		Serine		12.1	
		Hydroxyproline		10.9	
		Glycine		9.8	
		Alanine		8.7	
		Valine		7.9	
		Lysine		7.3	
		Threonine		6.2	
		Glutamic acid/glutamine		5.7	
		Cysteine		5.4	
		Phenylalanine		3.8	
		Isoleucine		3.7	
		Leucine		2.3	
		Proline		1.8	
		Tyrosine		1.0	
		Aspartic acid/asparagine		0.6	
		Methionine		0.3	
		Tryptophan		n.d.	
		Arginine		n.d.	
		Norvaline		n.d.	

* Percentage values are related to the quantification values determined by HPACE-PAD under consideration of linkage type distribution according to GC-MS; ** determined by Bradford assay; *** determined by HPAEC-PAD after hydrolysis; **** determined according [31].

The overall content of amino acids in F2 detected after hydrolysis was 1.03% ± 0.1% with histidine, serine, hydroxyproline, glycine, alanine, valin and lysine being the major monomers (Table 2). This result indicated the existence of a hydroxyproline-rich AGP [32]. The presence of ~10% Gly was not considered to be typical for AGPs, but in recent literature some reports have described such Gly-rich AGPs [33,34,35,36]. As it is usual for AGs we assume O-glycosylation via Hyp, Thr and Ser [28].

Enzymatic treatment of F2 with different enzymes followed by subsequent gel permeation chromatography on Superose^®^6 stationary phase indicated the formation of low molecular oligosaccharides (<9 kDa) beside the high molecular arabinogalactanprotein when *endo*-arabinanase and α-l-arbinofuranosidase were used, indicating the presence of longer α-linked l-arabinose side chains. Treatment with *endo*-β-1,6-galactanase resulted in the formation of low molecular weight oligosaccharides. In this experiment the main high molecular peak was still the very dominant peak, indicating that the polysaccharide backbone can not consist of β-1,6-linked galactose, but should be (according to the data from methylation analysis) a 1,3-linked galactose chain.

**Figure 3:**
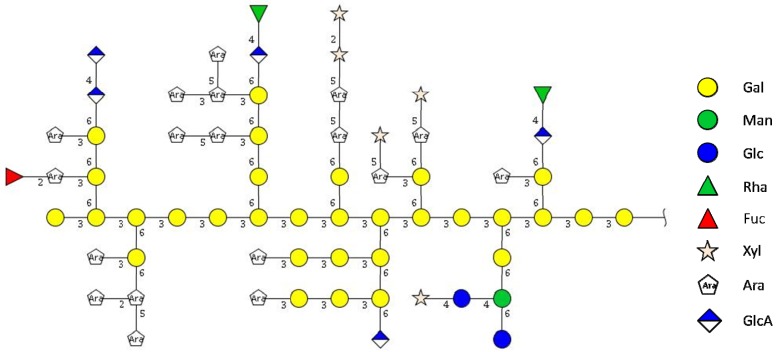
Proposed structure of the carbohydrate moiety of F2.

A higher degree of degradation was obtained when a mixture of *endo*-β-galactanase and α-l-arabinofuranosidase was used. This finding indicates that the arabinose residues should be linked directly to the galactose units. Treatment of F2 with β-glucuronidase liberated low amounts of low molecular material. Summarizing from these data a potential structure of the carbohydrate part of arabinogalactan protein F2 from *R. nigrum* can be made according Figure 3.

### 2.2. Antiadhesive Activity of F2 against H. pylori

**Figure 4:**
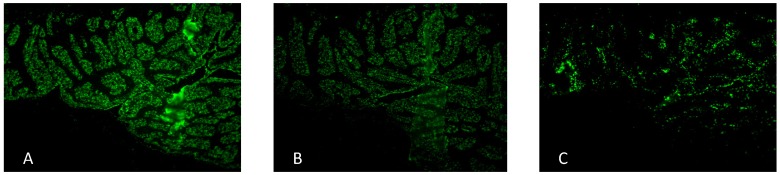
Representative fluorescence microscopy images of FITC-labelled *H. pylori* adhering to human gastric mucosa sections: untreated *H. pylori* control (**A**), arabinogalactan protein F2 from *R. niger* (1 mg/mL) (**B**). *H. pylori* pre-treated with acidic human milk oligosaccharides (positive control) (**C**), Images (magnification 100×) are equalized in brightness and fluorescence intensity and assessed by double blinded microscopic evaluation as well as by fluorescent imaging by ImageJ^®^ software.

For investigation of antiadhesive activities of F2 two different test assays were used: beside a semi-quantitative *in situ* adhesion system, based on gastric tissue sections from human biopsis [10,14] an *in vitro* flow cytometric assay with human gastric epithelial AGS cells and FITC-labeled bacteria was used to quantify potential antiadhesive effects [12]. Within *in situ* adhesion system on human gastric tissue the arabinogalactan protein F2 (1 mg/mL) exhibited about 25% of inhibition of bacterial adhesion to the host cells (Figure 4). 

**Table:** 

*H. pylori* pre-treated with	concentration	Blinded fluorescence microscopy evaluation *	rel. fluorescence area (%)
A untreated negative control	--	+ + + + +	100
B Arabinogalactan protein F2	1.0 mg/mL	+ + +	75
C Acidic human milk oligosaccharides (positive control)	1.0 mg/mL	+ +	13

* +++++ : strong adhesion, + weak adhesion.

**Figure 5:**
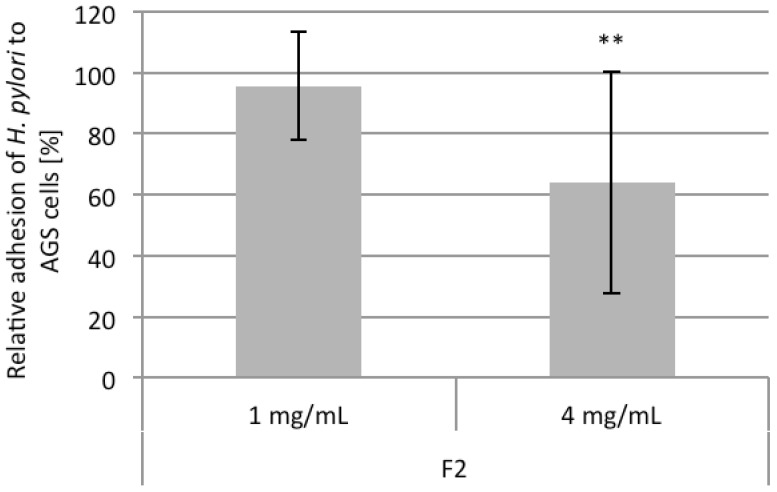
Relative adhesion (%, related to the untreated control UC) of FITC-labeled *H. pylori* to AGS cells after pretreatment of the bacteria with 1 and 4 mg/mL of arabinogalactan protein F2 from *R. nigrum*. Values are mean ± SD; ** *p* < 0.01 related to the untreated control (=100%).

Within the *in vitro* adhesion test system with flow cytometric evaluation antiadhesive activity of F2 (4 mg/mL) against pretreated *H. pylori* was detected with about 40% inhibition of bacterial adhesion (Figure 5). 

It seems interesting that higher concentrations of F2 (4 mg/mL) had to be used in this *in vitro* experiment compared to the experimental protocol used in the *in situ* assy (1 mg/mL), probably due to different expression of surface binding structures of host cells and tissue. As the fraction F2 was heterodispers and consisted of 3 subfractions (see GPC-data as described above) the different polymer fractions were tested and only the AGP from the highest molecular fraction was found to exhibit antiadhesive activity, in contrast to the other fractions with lower molecular weight.

The absence of cytotoxic effects of F2 against *H. pylori* was verified by an agar diffusion test, and no toxic effects were detected (0.5 to 4 mg/mL); in addition, no cytotoxic effects against AGS cells were recorded (data not shown). No inhibitory activity was observed during the pretreatment of AGS cells, which indicates that F2 should target only the bacterial cell surface. 

For investigation of the molecular targets of arabinogalactan F2 on the bacteria a semiquantitative dot blot overlay assay [15] was performed to pinpoint the respective bacterial adhesins blocked by the polymer. Therefore, putative ligands known to interact specifically with *H. pylori* adhesins were immobilized by spotting on PVDF membranes. A representative selection of ligands identified for *H. pylori* adhesins used for these experiments were: Le^b^- and H type I-conjugates (which interact specifically with BabA); sialyl-Lewis^a^, and laminin (known for interacting with SabA), and fibronectin (with a not-yet-determined bacterial adhesin affinity). In addition to the use of human serum albumin (HSA) and bovine serum albumin (BSA) as controls to exclude non-specific binding of *H. pylori* to spotted compounds on the membrane, 6'-sialyllactose also served as a control.

**Figure 6:**
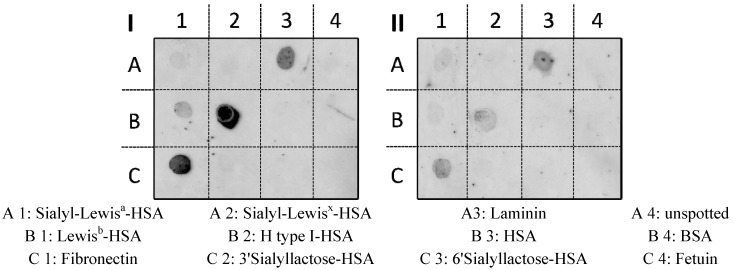
Representative images of the adhesion of FITC-labeled *H. pylori* wild-type strain J99 to immobilized ligands on PVDF membranes: (**I**) untreated control and pretreated bacteria with (**II**) arabinogalactan protein F2 (4 mg/mL). (Neo)glycoproteins spotted on PVDF membranes (1 μg per spot) were overlaid with FITC-labeled *H. pylori* and adherent bacteria were detected by fluorescence imaging. The respective locations of spotted (neo)glycoproteins are indicated below.

As expected, high binding affinity of untreated *H. pylori* to the immobilized Le^b^, H type I as ligands for BabA, to fibronectin and laminin as was obvious; relatively low, but distinct binding was observed for the immobilized sialylated Le^a^ and Le^x^ as ligands for SabA (Figure 6).

No adhesion to spotted 3'-sialyllactose-HSA conjugate was observed when bacteria were preincubated with 3'-sialyllactose. Pretreatment of *H. pylori* with arabinogalactgan protein F2 (4 mg/mL) reduced binding to the respective immobilized HSA-conjugates of Le^b^, H-1, laminin and fibronectin, whereas interaction with s-Le^a^-HSA, s-Le^x^-HSA and laminin was not influenced.

Therefore, it can be deduced that the arabinogalactan protein interacts with BabA and an unknown fibronectin-binding adhesin, but does not influence the binding to the SabA and HpaA related binding sides. This also explains the reduction of *H. pylori* adhesion to gastric epithelial cells under *in vitro* and *in situ* conditions. This finding that F2 does not interact with SabA (SabA is the bacterial hemagglutinin of *H. pylori* with strong binding affinity to NeuAc2a2-3Gal) was confirmed by hemagglutination assay using human blood (0-). In this experiment acidic human milk oligosaccharides served as a positive control, because they contain structures of α-2,3-linked sialic acid motifs together with fucosylated oligosaccharides and should reduce *H. pylori*-mediated hemagglutination. This positive control revealed a reduction of agglutination titer within 1:2 serial dilution assays of 2.5 ± 0.8 titer steps at 1 mg/mL test concentration while F2 (1 to 4 mg/mL) did not influence hemglutination. 

### 2.3. Binding of Radiolabeled F2 to Different Strains

Radioimmunoassays were performed to analyze whether the reduced binding of *H. pylori* to H-1 and Le^b^ after the bacteria is pretreated with arabinogalactan F2 is mediated by specific interaction with BabA. F2 was radiolabeled [37], purified, and fractionated by PD-10 gel permeation. The maximal radioactivity was obtained for three fractions (3,4,5) representing the radiolabeled high molecular glycoproteins. Le^b^-HSA and sLe^x^-HSA were also iodinated in a similar way. Binding experiments against a variety of different *H. pylori* strains revealed strong interactions of fractions 3, 4, and 5 with all strains. (note: For this experiment we can not provide statistical data, but only single experiments because of limited amount of radiolabeled material)

**Figure 7:**
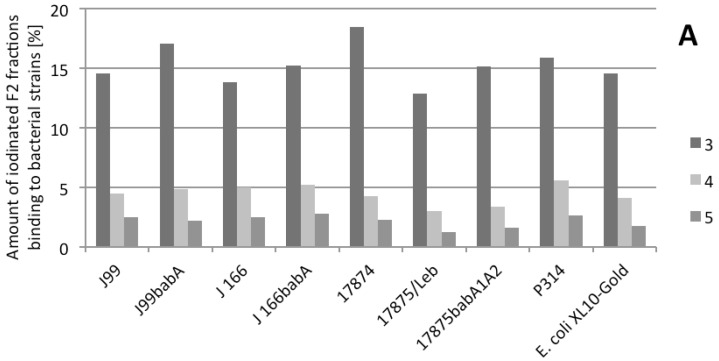
Binding of radiolabeled fractions of *R. nigrum* arabinogalactan protein F2 (subfractions FE3, 4 and 5) to different *H. pylori* bacterial strains and *E. coli* reference strain.

Although fraction 3 (the highest molecular weight fraction) exhibited the strongest interaction, fractions 4 and 5 also interacted with the bacteria (Figure 7).

In principle, similar F2 binding was observed for all strains tested (e.g., for the wild type strain J99 (BabA, SabA positive), as well as for the J99 BabA deletion mutant). The same was true for wild type J166 (BabA, SabA positive) and its respective BabA mutant. Strain 17874 (BabA neagtiv, SabA positive), strain 17875/Le^b^ (BabA positive, SabA negative), BabA deletion mutant 17875*babA1A2* (BabA negative, SabA positive), and strain P314 (BabA positive) also interacted with F2 fractions. Therefore no specifity for BabA interaction can be seen as no difference between the binding to strains J99 and J166 and to the respective *babA* deletion mutants was observed. Moreover, the interaction of F2 does not appear to be specific for *H. pylori*, because the labeled bacteria also bound to the *E. coli* control strain.

### 2.4. Competiton of Arabinogalactan Protein F2 with Le^b^ and sLe^x^ Ligands

**Figure 8:**
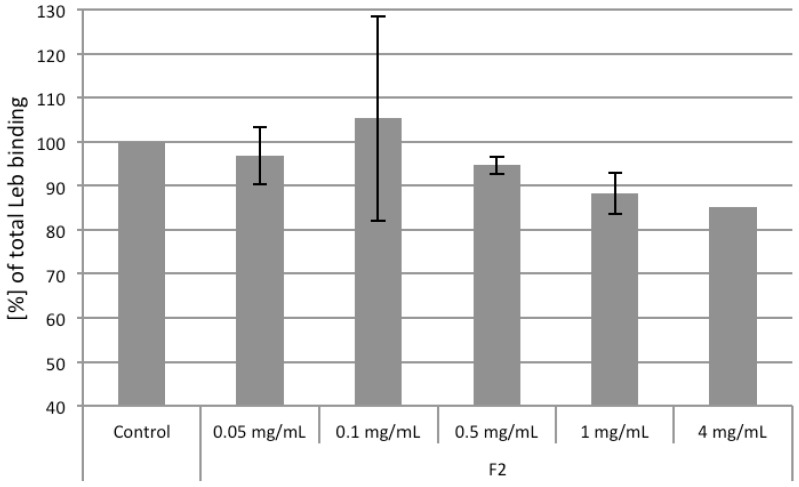
Influence of *R. nigrum* arabinogalactan proteins F2 on *H. pylori* strain 17875/Le^b^ binding to Le^b^-HSA. Data from two independent assays (4 mg group only with one assay, due to limited substance amount) are related to the Le^b^-HSA binding of the control (only 17875/Le^b^ together with Le^b^, = 100% Le^b^ binding). Bar of the control group: SD, F2 test groups: range between the two experiments.

To investigate whether F2 influences the binding of *H. pylori* to Le^b^, we coincubated labeled Le^b^-HSA conjugate with unlabeled F2 in the presence of the strong Le^b^ binding strain 17875/Le^b^. F2 inhibited the binding of 17875/Le^b^ to Le^b^-HAS very moderately (Figure 8). 

Inhibitory effects of approximately 10% to 18% were achieved in the concentration range of 1 to 4 mg/mL. Although these effects are not seem to be very strong, a clear inhibition of F2 towards 17875/Le^b^ was visible, which supports also the about 20% inhibition of bacterial adhesion observed in the different functional assays. 

To see whether these effects also occur with the *H. pylori* strains J166 and P314, which are known to have an approximatly 100-fold lower affinity for Le^b^ compared with 17875/Le^b^ (J166: *K*_a_ 4.34E+09 M^−1^; P314: *K*_a_ 5.57E+09 M^−1^) [38], we also tested these strains. Concentrations of F2 up to 4 mg/mL did not reduce the binding of Le^b^ to strain P314 and J166 (data not shown). 

To investigate the influence of F2 on *H. pylori* binding to sialyl-Lewis^x^, radioimmunoassay investigations were performed using strain 17875*babA1A2* because it binds sLe^x^, but not Le^b^ [19]. The 17875/Le^b^ strain served as blank, because it does not bind sLe^x^ [19]. As expected from dot blot overlay experiments and hemaglutination assay no binding of F2 was observed, again indicating no interaction of the arabinogalactan protein with SabA. 

### 2.5. Differential Gene Expression

To determine the influence of F2 on the gene expression of bacterial adhesins and virulence factors, and especially on the potential correlation of adhesion with the expression of virulence factors, *H. pylori* was incubated with arabinogalactan F2. Using 23S rRNA as an endogenous control, we monitored the influence of F2 on several OMPs (*babA*, *alpA*, *alpB*, *hopZ*, *oipA*, and *hpaA*). Moreover, the gene encoding *α-*1,3-fucosyltransferase (*fucT*), which is involved in catalysis of the Lewis^x^ trisaccharide (a major component of *H. pylori* lipopolysaccharides), was also included in the study [39]. We also assessed genes encoding *vacA* and *cagA*, as well as *cagL* and *cagα* (encoded in the cagPAI pathogenicity island for the type 4 secretion system TFSS), the metalloenzyme urease (*ureA*), and a regulator for the transport of urea by an acid-gated urea-channel (*ureI*) [40].

**Figure 9:**
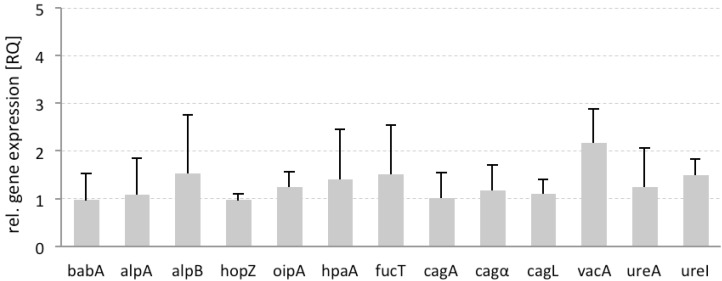
Differential gene expression of *H. pylori* pretreated with arabinogalactan protein F2 from *R. nigrum*. Endogenous control: 23S rRNA. Data are related to untreated control (UC) *H. pylori* in liquid growth medium (RQ = 1), with n = 2 replicates from three independent experiments (mean ± SD).

For this study, the gene expression of untreated *H. pylori* was set as the reference (RQ = 1) in relation to the gene expression of pretreated bacteria. Preincubation with F2 did not change the gene expression of the bacterial adhesins, also the expression of genes encoding all virulence factors did not change significantly, with vacA showing a tendency toward increase (Figure 9). These data indicate that the interaction of F2 with the adhesins does not induce an automatic feedback mechanism in the bacterial cell that is associated with increased or dramatically decreased functionality.

### 2.6. Discussion

From detaffed seeds of *R. nigrum* an arabinogalactan protein (AGP) was isolated and characterized concerning the structural features. In many cases AGPs are found in seed embryonic tissue, were they exert central functions during embry development, germination and xylem formation [41]. AGPs are mostly mixtures of different proteins and peptides [33] linked to complex arabinogalactan polysaccharides, which leads to a deactivation of protein functionality. The protein activity might again be restored after reconstitution of the protein from the carbohydrate core. In case of F2 from *R. nigrum* the broad molecular weight dispersity seems also to point towards the existence of various peptides within a more or less constant arabinogalactan moiety. Further MS studies on domain-containing proteins should be performed to elucidate potential sequence homologies to prtoteins described in literature for seed development.

AGPs are getting more and more into the focus for pharmacological activities, e.g., immunoactivation, influence on cellular signal transduction *etc.* This report on antiadhesive activities is the first study, which indicates that AGPs can also influence bacterial adhesion. This aspect might be interesting for further cytoprotective strategies to use black currant fruit and seeds for food products for establishment of preventive strategies against stomach infectionas, caused by *H. pylori.* This has also to be seen within the fact that standard eradication therapy of *H. pylori* has been successfully implemented, but considerably high recurrences rates are documented. The annual reinfection rate is quite low in developed countries (3%), but considerably higher in developing countries (13%) [42,43]. In addition, the increasing antibiotic resistance of *H. pylori* is being monitored. Therefore, advanced molecular targets must be pinpointed for future *H. pylori* treatment. The development of antiadhesive compounds that interfere with OMPs and block bacterial adhesion might be an interesting approach for prevention [6,44,45] Most *H. pylori* infections occur during the first 2 to 5 years of life [8]; in principle, the development of such antiadhesive compounds toward products for use in food or health products might help to prevent very early infection in children [9]. After antibiotic treatment, some patients experience recurrence of the infection after several months (a problem mainly in developing countries, such as South America and Asia), and it is possible that these patients might benefit from the use of such compounds in food supplements to be used during and after antiobiotic eradication therapy. In this context, translational developments of antiadhesive plant extracts are of interest.

The antiadhesive activity of the here described arabinogalactan protein can be compared to other antiadhesive polysaccharides as for example highly acidic rhmanogalacturonans from liquorice [13] or Okra fruits (*Abelmoschus esculentus)* [15]. Especially the latter was shown to be inhibit BabA, and it seems interesting that also this inhibitor does not act directly against BabA, but changes the binding-capacity of the bacterial adhesion to Le^b^ by interaction with surface structures in vivinity to BabA. This seems similar to the effect determined for F2. Additionally the rhamnogalacturonan from Okra seems toinfluence also the binding of SabA to sialylated Le^x^ which is not the case for F2. This can be interpretated that the highly charged rhmanogalacturonans might have higher affinity to the adhesins with affinity to negatively charged sialylated ligands. F2, a nearly neutral AGP, does not influence SabA, which might be due to the missing charge. This hypothesis could be proven by further investigation by using other neutral polysaccharides described as antiadhesive polymers, e.g., galactans from *Kingella kingae* [6].

Concerning the structural features necessary for the bioactivity it is known that non-protein associated arabinogalactan, as it can be obtained from Larix sp. as a pure polysaccharide does not inhibit *H. pylori* adhesion [46]. From this point we assume that the intact AGP as a fixed combination o glycan and polypeptide is needed for the antiadhesive effects.

The chemical synthesis and optimization of specific inhibitors of the major adhesins BabA and SabA might be possible, and might be a promising tool for future pharmaceutical and clinical development. For optimized *in silico* definition and chemical synthesis of inhibitors with specific activity toward the active center of these lectin-like proteins, more detailed investigations regarding the molecular and physical characteristics of the adhesins are necessary; specifically, no protein crystal data have been published at this time.

Such specific BabA inhibitors with an underlying structure-activity relationship have recently been described for the class of N-phenylpropenoyl amino acid amides [47]; however, specific blocking of BabA only leads to a 20% to 30% reduction of bacterial adhesion. Because other adhesins (SabA, Alp, OipA, HopZ, *etc.*) are sufficient to achieve successful adhesion, the inhibition of BabA alone does not strongly minimize infection of the stomach cells. Because F2 from *R. nigrum* acts mainly over BabA inhibition this explains the moderate inhibition effects of about only 20% within the different test assays. 

**Table 2:** Primer sequences for *H.pylori* (Qiagen). F = Forward Primer; R = Reverse Primer, P = QuantiProbe (label = FAM); * = modified nucleotide.

Primer	Gene/locus tag (Ref.)		Sequence, 5' → 3' orientation
23S rRNA	jhpr2 [44]	F	CCTTAGATTTACGGCGGATACA
		R	CAGTGGTAGAAGAGCGTTCATA
		P	TCACTTCATACCGCTCC
*AlpA*	jhp0848 [44]	F	GCTCACTAAAAACACCATT
		R	CATGCTTCTCACCTGATTGTTG
		P	TCCAACCAAGCTAACGC
*AlpB*	jhp0849 [44]	F	GAGTCAAAACATCAGCAAGA
		R	ACTGAGCTGGTTGGAGAGATT
		P	GACAACAACACCACGA
*BabA*	jhp0833 [44]	F	GATTTGTTATCGTTTGTCCTAC
		R	AGGCTTAGCGGGACTTTT
		P	GTTGATGG*GTTGTTGC
*HopZ*	jhp0007 [44]	F	CCAAGAAATCGTAACGCAAG
		R	TGTTTTGAGCGAAAGCCTATC
		P	TCCTTACACCTCTGCT
*OipA*	jhp0581 [44]	F	ATAAGCGAGCGTGTCAAGAA
		R	ATGCCAATCACAAGCCCTGAA
		P	GAAAGAAGG*GTAAAAGG
*HpaA*	jhp0733 [44]	F	CAAACCAGTGGAGAATAATAAC
		R	GGATAGCAGCGATAAAGACGAT
		P	CCATTCATAGCGACAG
*FucT*	jhp0596 [44]	F	TGCAAGTATCTCACGTAATCAA
		R	CTCAAGGCTATGGCTATGTAAC
		P	TGG*GAATGGTGTGGCT
*CagA*	jhp0495 [44]	F	TGGCAGTGGGTTAGTCATA
		R	CCTGTGAGTTGGTCTTCTTTGT
		P	AGGTGGTGAGAAAGG*GA
*CagL*	jhp0487 [44]	F	CTCGATTTTCAGCTTCCC
		R	TCAATCCCTTAGACCAAAAGACT
		P	ATTCCGCATTGTTGCT
*Cagα*	jhp1344 [44]	F	GAGACAAGCTCCATGAGA
		R	ACCCCCGGTTCATAAAGACT
		P	ACTTATTCTCCCACTTGC
*VacA*	jhp0819 [44]	F	AAACGACAAGAAAGAGATCAGT
		R	CCAGCAAAAGGCCCATCAA
		P	CAATAGCAACACAGAGG
*Ure I*	jhp0066 [44]	F	AGTGTTGATCGCTACGAATAAG
		R	AGCGACTGGGTTATTGTTTGG
		P	AGTGTGGTTGATAGCGG
*UreA*	jhp0068 [44]	F	TTGCCTTCGTTGATAGTGATG
		R	CTGATGGGACCAAACTCGTAA
		P	AACAACTCACCAGGAA

Gene expression analysis with F2-treated *H. pylori* clearly indicated that inhibition of bacterial adhesion by this AGP does not necessarily lead to changes in gene expression of adhesion or virulence factors. This implies that the status of the adhesion factors is not automatically triggered with the expression of bacterial virulence. On the other side it is known [48,49] that interaction of mucins with *H. plyori* can induce expression of genes important for the pathogenicity of *H. pylori* (*babA*, *sabA*, *cagA*, *flaA* and *ureA*) and at least Muc-1 ligands seem to be coregulaters in bacterial responses to ligand binding.

Another explanation for understanding the mode of action of F2 and other antiadhesive polysaccharides (as e.g., described in [15]) could also be a bioadhesive affinity to the cell surface, leading to changes in surface charge, similar as it has been described for antiadhesive and anti-biofilm galactans [6] From recently published data with magnetic nanoparticles with defined charges it is known [15] that the binding of polysaccharides to the surface of *H. pylori* depends mainly on the molecular charge. Also this would support the hypothesis that the antiadhesive compounds act via changing the surface charge which agains seems to influence the functionality of BabA.

Another problem has to be discussed, namely the phenomenon that under *in vivo* conditions most *H. pylori* will be located in or on the gastric mucus phase. From previous investigations [unpublished data] we know that polysaccharides from *R. nigrum* have no affinity to rat stomach epithelia or exert mucilaginous effects to intact tissue; this has been proven within an *ex vivo* adhesion bioassay. We assume that F2 will only interact with free floating bacteria in the stomach liquid, or bacteria at a very early stage after entry into the organism. On the other side *H. pylori* in the stomach are undergoing a continous docking and undocking process, which means that antiadhesive compounds as F2 could shift the equilibrium to the free floating, unbound form.

It is interesting that F2 does not interact directly with the active sites of BabA, but with still-unknown surface structures in the vicinity of the adhesins. This means that the inhibiting effects of F2 may not be affected by the mutation of the adhesins. 

It must be kept in mind that antiadhesive compounds would likely have to be used longterm in terms of preventive treatment, and undesired effects on intestinal flora and the intestine might occur, especially due the low degree of specifity. 

It might be interesting to monitor also the influence of antivirulence drugs, especially of antiadhesive compounds on the development of bacterial resistance. In case the mode of action of such entry-inhibitors as e.g., F2 is quite unspecific the development of resistant bacteria might occur not or very slowly. On the other side more and more reports are published on development of resistance against anti-virulence drugs [50,51,52] describe evolution of resistance to *quorum-sensing* inhibitors, and it has to be monitored if similar changes will be observed also for antiadhesive compounds.

The use of crude plant extracts may be a valuable tool for future developments, also keeping in mind how economical it can be to obtain such preparations. It has to be realized that quite high doses of F2 had to be used for inhibition of bacterial adhesion. This is in accordance to literature published on other antiadhesive candidates against *H. pylori*. For example the clinical testing of sialyllactose against *H. pylori* [8] in humans was performed with gram-doses. From the economical point of view, this makes no sense [53]. In contrast to that the black currant extract can be produced easily from the plant material without big economical impact. 

The data presented here suggest sufficient *in vitro* antiadhesive activity, which must be proven in future animal and clinical studies. Nevertheless, the above documented results demonstrate that antiadhesive compounds like the arabinogalactan do not enhance *H. pylori* virulence at the mRNA level. Once again, these data enhance the potential of antiadhesive compounds for future applications for the prophylactic control of *H. pylori* infections within a cytoprotective strategy. However, due to the genetic heterogeneity of *H. pylori* strains and the resulting complexity of the adhesion mechanism, further investigations with additional clinical isolates are suggested.

## 3. Experimental

If not stated otherwise all chemicals were purchased by Sigma (Deisenhofen, Germany) and from Merck (Darmstadt, Germany) in analytical quality. 3'-Sialyllactose (NeuAcα_2-3_Galβ_1-4_Glc), fluoresceinisothiocyanate isomer I (FITC), chloramine T, and sodium bisulfite were purchased from Sigma Chemicals (St. Louis, MO, USA). The total fraction of acidic human milk oligosaccharides was obtained from the Danone Research Centre for Specialised Nutrition (Friedrichsdorf, Germany). 

### 3.1. Isolation of R. nigrum Arabinogalactan Protein (AGP)

*R. nigrum* L. seeds were obtained from Glaxo SmithKline Beecham (Herrenberg, Germany) and were identified by the comparison with reference drug material. A voucher species is deposited in the archives of IPBP, University of Münster “Johannisbeersamen, BMII/2001”. 

Seed material from *Ribes nigrum* L. (500 g) was crushed under liquid nitrogen to a fine powder. Defatting was performed by Soxhlet extraction with acetone for 24 h (yield 425 g). Polysaccharides were extracted three times with 4 L of water at 8 °C under strong stirring. After centrifugation the volume of the combined extracts was reduced under vacuum (max. 40 °C) and the resulting concentrate (1 L) precipitated in ice-cold 96% ethanol (4 L) of The precipitate was isolated by centrifugation (924 *×g*), dissolved in water (500 mL), dialyzed (Cellulose membranes, MWCO 3500 Da, Roth, Karlsruhe, Germany) and lyophilized to yield 1.1%, related to the starting seed material. F2 was isolated by AEC using a DEAE Sephacel^®^ (General Electric, Munich, Germany) column (23 × 3.5 cm) in the phosphate form and elution by a step gradient of deionized water, sodium phosphate buffers pH 8.0, ion strength 0.1-, 0.25-, 0.5-, 1 mol/L, flow 78 mL/h, fraction size 9 mL. Carbohydrate-containing fractions were pooled, concentrated under vacuum, dialyzed and lyophilized. F2 was isolated from the 0.1 mol/L eluate (0.3%). 

### 3.2. Glycoprotein Analysis

All methods used for analysis of F2 AGP are described in [33,34]. 

### 3.3. Bacteria and Growth Conditions

*Helicobacter pylori* ATCC 700824 (strain J99, identification for quality control by PCR for vacA and cacA genes) was cultivated for two or three passages to minimize the risk of phase-variable switching of OMP genes. Cultivation was performed according to [10]. We used strains CCUG17874; CCUG17875/Le^b^ [54]; CCUG17875*babA1A2*, a babA1::kan babA2::cam mutant of CCUG17875 [19]; J99*babA*, a J99babA::cam mutant of J99 [19]; J166 [55]; J166*babA* (J166*babA*:rpsl CAT CR); and strain P314, an isolate from San Juan de Miraflores (Lima, Peru) [38], in addition to an *E. coli* control strain (XL10-Gold, Strategene, Santa Clara, CA, USA). Bacteria were grown on Brucella agar supplemented with 10% bovine blood and 1% IsoVitox Enrichment (Svenska LABFAB, Ljusne, Sweden) for 36–48 h at 37 °C, under 10% CO_2_ and 5% O_2_ before harvest in phosphate-buffered saline (PBS) containing 0.05% Tween 20% and 1% bovine serum albumin (BSA; Saveen Werner AB, Sweden). Labelling of bacteria, *Helicobacter pylori* adhesion assays, hemagglutination assay, dot blot overlay assay, gene expression analysis.The respective assays were performed as described by [9]. Primer sequences for gen expression analysis are displayed in Table 2.

### 3.4. Cell Culture

Human adherent gastric adenocarcinoma epithelial cells (AGS, ATCC CRL-1730) were kindly provided by Prof. W. Beil (Medizinische Hochschule Hannover, Hannover, Germany). Cells were grown as described previously [12].

### 3.5. Radiolabeling of Arabinogalactan F2 and Glycoconjugates

Radiolabelling [37] of F2 was performed as described by [16] using 0.2 mg of F2 dissolved in 50 µL buffer. The same procedure was applied to Lewis^b^ and sialyl-Lewis^x^ (2 μg) [16].

### 3.6. Analysis of Binding Properties of F2 towards Different H. pylori Strains by Radioimmunoassay

^125^I-labeled fractions of F2 were diluted 10-fold with blocking buffer (PBS containing 1% BSA and 0.05% Tween 20). The tested strains were *H. pylori* strains J99, J99*babA*, J166, J166*babA*, CCUG17874 (17874), CCUG17875/Le^b^ (17875/Le^b^), CCUG17875*babA1A2* (17875*babA1A2*), and P314; and *E. coli* XL10-Gold. A bacterial suspension adjusted to OD_600_ 0.1 per mL was used. Ten microliters of radiolabeled sample were added to 1 mL of bacterial suspension. After mixing, samples were incubated for 17 h at RT on a rocking table. Bacteria were pelleted by centrifugation (16,058 *×g*, 13 min) and the supernatant was separated from the pellet. Both supernatant and pellet were collected into plastic tubes and measured with a gamma counter (counts per min) for 10 min. Differences in radioactivity between the pellet and the supernatant reflect the ability of bacteria to bind to the test compounds.

### 3.7. Inhibitory Effects of F2 on Lewis^b^ Binding of H. pylori

An OD_600_ 0.1 suspension of two different bacterial strains (J166, P314) was prepared in citrate buffer, pH 5. 500 μL of each suspension was transferred to reaction tubes. For the third tested strain, 17875/Le^b^, the suspension was diluted to an OD_600_ of 1.3 × 10^−4^ (calculated) by mixing with *H. pylori* strain 17874 (OD_600_ 0.1/mL) and blocking buffer (PBS with 1% BSA (Cohn fraction V, protease-free, Saveen & Werner AB, Limhamn, Sweden). Addition of 17874, a strain that does not bind Le^b^ [56] was necessary to create a visible pellet.

For the CCUG17875/Leb strain, which has a high binding affinity (K 3.9E+11 M^−1^) [38], the bacterial suspension was serially diluted to an OD 600 of 1.3 × 10^−4^ (calculated) in order to meet an assumption of an analysis of binding experiment that only a very small fraction of the radioligand binds both specifically and non-specifically [57].

Next, sample solutions (450 µL) with different concentrations were added to the reaction tubes. Finally, radiolabeled Le^b^-HSA (50 µL, 0.2 ng) was pipetted carefully into each reaction tube. Samples with bacteria and radiolabeled Le^b^-HSA alone served as controls (no inhibitor added), and samples with radiolabeled Le^b^-HSA and *H. pylori* strain 17874, which does not bind Le^b^, served as a blank. After mixing, samples were transferred to a rocking table and were incubated and measured.

### 3.8. Inhibitory Effects of F2 on Sialyl-Lewis^x^ Binding of H. pylori

These experiments were performed similarly to those described above regarding Le^b^ binding; however, blocking buffer, which contains periodate-treated BSA that lacks sialic acid, was used instead of citrate-phosphate buffer. The use of this pre-treated BSA is necessary during experiments concerning the SabA adhesin of *H. pylori* because the sialic acid structures in BSA may interfere with the adhesin, leading to false results [38]. *H. pylori* strain 17875*babA1A2* was used to determine SabA-mediated binding, and 17875/Le^b^ served as a negative control. An amount of 1 ng/mL iodinated sLe^x^ was used for these experiments.

### 3.9. Statistics

Statistical results were obtained by the use of SPSS^®^ statistics software volume 20 (IBM, Armonk, NY, USA). Results were expressed as mean value (MV) ± standard deviation (SD). After Levene’s test on variance homogeneity, analysis was performed using one-way analysis of variance (one-way ANOVA). If results revealed significant differences between group mean values, then groups were compared using the Dunnett test (2-sided), with *p* < 0.05 considered statistically significant (*) and *p* < 0.01 considered highly statistically significant (**).

## 4. Conclusions

The *R. nigrum* arabinogalactan protein can be assessed as follows: the compound can be easily manufactured by simple fruit extraction followed by galenical formulation of the hydrophilic compound; the arabinogalactan must be doses (in the mg range) to affect bacterial adhesion; the compounds are not bioavailable in the systemic compartment because of their high molecular weight and hydrophilicity and toxicity against human cells is not expected. Infection studies must evaluate whether the inhibition of a single adhesin can effectively prevent bacterial adhesion and lead to reduced infection rates.

## Abbreviations

AEC: anion exchange chromatography
AGP: arabinogalactan protein
BabA: blood group binding adhesion
F2: arabinogalactan protein fraction from *R. nigrum* after AEC
FITC: fluorescein isothiocyanate
MALLS: multi angle laser light scattering
OMP: outer membrane protein
RT-PCR: real-time polymerase chain reaction
SabA: sialic acid binding adhesion
